# Formation of Fractal Dendrites by Laser-Induced Melting of Aluminum Alloys

**DOI:** 10.3390/nano11041043

**Published:** 2021-04-19

**Authors:** Alexey Kucherik, Vlad Samyshkin, Evgeny Prusov, Anton Osipov, Alexey Panfilov, Dmitry Buharov, Sergey Arakelian, Igor Skryabin, Alexey Vitalievich Kavokin, Stella Kutrovskaya

**Affiliations:** 1 Department of Physics and Applied Mathematics, Stoletov Vladimir State University, Gorkii Street, 600000 Vladimir, Russia; kucherik@vlsu.ru (A.K.); samyshkin@vlsu.ru (V.S.); osipov@vlsu.ru (A.O.); buharov@vlsu.ru (D.B.); arak@vlsu.ru (S.A.); sk-ig@vlsu.ru (I.S.); 2 Department of the Functional and Constructional Materials Technology, Stoletov Vladimir State University, Gorkii Street, 600000 Vladimir, Russia; prusov@vlsu.ru (E.P.); aapanfilov@vlsu.ru (A.P.); 3 ILIT RAS Branch of FSRC Crystallography and Photonics RAS, 140700 Shatura, Russia; 4 Westlake University, 18 Shilongshan Road, Hangzhou 310024, China; a.kavokin@westlake.edu.cn; 5 Institute of Natural Sciences, Westlake Institute for Advanced Study, 18 Shilongshan Road, Hangzhou 310024, China; 6 Russian Quantum Center, Skolkovo IC, Bolshoy Bulvar 30, bld. 1, 121205 Moscow, Russia

**Keywords:** alloys and composite materials, laser action, boron carbide, clusters

## Abstract

We report on the fabrication of fractal dendrites using laser-induced melting of aluminum alloys. We target boron carbide (B${}_{4}$C), which is one of the most effective radiation-absorbing materials characterized by a low coefficient of thermal expansion. Due to the high fragility of B${}_{4}$C crystals, we were able to introduce its nanoparticles into a stabilization aluminum matrix of AA385.0. The high-intensity laser field action led to the formation of composite dendrite structures under the effect of local surface melting. Modelling the dendrite cluster growth confirms its fractal nature and sheds light on the pattern behavior of the resulting quasicrystal structure.

## 1. Introduction

Aluminum alloys (AAs) are promising for various industrial application due to a unique combination of the low volume density, high specific strength, corrosion resistance, and thermal conductivity. High-strength aluminum alloys are significantly superior to low-carbon and low-alloy steels as well as pure titanium in terms of strength-to-density and yield-to-density ratios. According to these criteria, they approach steel alloys of higher strength and titanium alloys [1]. To further improve AA mechanical properties, surface fabrication technologies such as ion implantation, plasma nitriding, and field assisted sintering technique (FAST) [2,3,4] are employed. Despite the fact that laser processing opens up various possibilities to locally modify metal surface properties [5,6], aluminum and its alloys are hardly sensitive to a laser field due to the combination of high values of reflectivity, thermal conductivity, and heat capacity [7]. Laser processing of AAs might be accompanied by the development of metallurgical microporosity. This microporosity is of gas and shrinkage nature [8], which is characteristic for casting of aluminum in general. It is important to note, however, that the skin-thickness laser processing does not allow us to reach the conditions for the evolution and migration of micropores due to the lack of a macroscale molten pool and, then, bulk crystallization process [9]. The surrounding atmosphere plays a significant role within AA processing that mostly leads to the formation of undesirable phases [10]. Thus, the hydrogen appearance motivates hydrated aluminum oxide growth, which generally reduces the relative density of the manufactured composite matter [11]. On the other hand, the nitrogen media initiates Al–N chemical bonding, which makes the whole complex quite fragile [12]. In this context, laser surface modification of AAs doped with microelements is of considerable interest from the point of view of increases in the resulting strength and improvements in the temperature characteristics of the composite material. Boron carbide (B${}_{4}$C) is a suitable admixture candidate, being an effective radiation-absorbing material that combines desirable mechanical properties such as alloy strength and heat resistance even under high temperatures and a low coefficient of thermal expansion [13]. Materials of such chemical compositions are characterized with a dendrite structure formed in a laser molten pool [14]. Their specific morphology is strongly dependent on the parameters of the laser action [15]. Fractal clusters are characterized by a much greater sedimentation stability compared to bulk 2D or 3D ones. The density of elements of a fractal cluster decreases from its center to the periphery. This means that there is almost no interfacial layer between the surrounding media and fractal structure. The transition from the center of the fractal cluster to the mother alloy is smooth. This specific feature makes fractal clusters unique and attracts a significant interest to them. This work is aimed at the study of laser-induced local modification of the aluminum alloy grade of AA385.0 embedded with B${}_{4}$C microparticles in the atmosphere. We show that a high-energy pulse regime of laser action with an energy of up to 5 J per pulse induces the formation of two types of fractal clusters evolving during the crystallization process. Their morphology is highly sensitive to the thickness of a locally melted surface. The observed phenomena of anisotropic cluster growth are in a good agreement with the results of our modelling.

## 2. Materials and Methods

The foundry of Al–Si alloy of AA385.0 grade according to the Aluminum Association nomenclature was used as a matrix for the composite production. The powdered boron carbide F150, 75–100 microns in particles size, was used as an exogenous reinforcing component. The AAs were purchased from United company Rusal (Krasnoyarsk, Russia),and the B${}_{4}$C powder from Zaporozhabraziv PJSC (Zaporizhia, Ukraine) was of analytical grade and was used without further purification. The full fraction composition were dosed according to the calculation of the charge for a melting volume of 200 g based on the nominal content of reinforcing particles in the composite at 5.0 wt%. Heating up to the melting point was provided by an electric resistance furnace in alundum crucibles. To increase the wettability of the B${}_{4}$C powder inside the melted matrix, titanium powder at 1.0 wt% was added before rolling into the aluminum foil. The matrix alloy was overheated up to 850 ${}^{\circ }$C; then, the slag was removed and a suspension of the boron carbide powder was fed to the melt surface under constant stirring with a four-bladed stainless steel impeller at a speed of 300 rpm for 5 min. After that, the slag from the melt surface was again removed and the resulting composite suspension was poured into a vertical copper mould at a temperature of 750 ${}^{\circ }$C in order to produce ingots of 20 mm diameter and 100 mm height. Samples were cut off from the bottom side at a distance of 15 mm from the obtained ingots to conduct high-energy laser experiments on the surface transformation and cluster growth.

To induce the laser–alloy interaction, we used milisecond laser pulses generated by an YAG:Nd solid laser with a central wavelength of 1.06 m, a pulse duration of 2.5 ms, a repetition rate of 3.5 Hz, and variable pulse energy up to 50 J. The laser spot size was about 800 m; however, in our setup, the laser beam was employed in a scanning regime on a rectangular area with a 1/4 overlap. For the detailed study of formed clusters, we performed scanning electron microscopy (SEM) using FEI Titan${}^{3}$ with a spatial resolution of up to 2 nm and Quanta 200 3D with EDAX column with a spatial resolution of up to 7 nm.

## 3. Results

### 3.1. The Surface Modification under Laser Action

**Figure 1:**
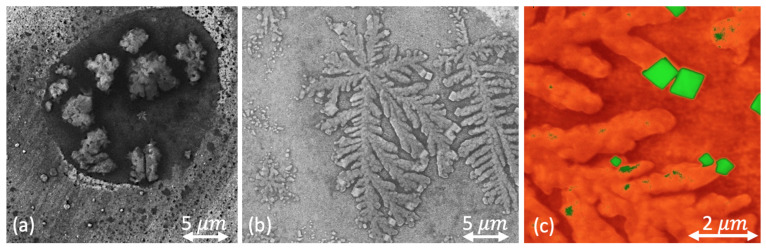
SEM images of cluster formation on an aluminum alloy surface under the effect of laser action: (**a**) a skeleton-type cluster, formed during local surface transformation at a pulse energy of 1 J; (**b**) a dendrite-type cluster, formed from a melted pool at a pulse energy of 5 J; the panel (**c**) combines the SEM-image and the results of an EDAX element analysis in order to make the B${}_{4}$C accumulation visible (B${}_{4}$C component marked by green while the alloy matrix is depicted with an orange color). One can see mostly cubic lattice formation of B${}_{4}$C at the ends of the branches.

To provide surface processing of the aluminum alloy composite, the periodic set of NIR laser pulses with a fiber delivery of radiation was used. Under the effect of laser pulse action with τ = 2.5 ms pulse duration and a wavelength of 1.06 m, the temperature at the laser spot center of a r=400 m radius can be found [16] for the case of a Gaussian beam using the following equation:

(1) $$T=\frac{2q\left(1-R\right)\sqrt{\alpha \tau }}{c\sqrt{\pi }}+T_{o},$$

where $q=\frac{E_{p}}{\pi r^{2}\tau }$, $E_{p}$ is a pulse energy (that has been measured experimentally and amounts to either 1 J for the solid surface transformation or to 5 J for the melted surface action), *R* is the aluminum reflection coefficient, *c* is the heat capacity, $T_{0}$ is the starting temperature, and α is the thermal conductivity. In the our experiments, the temperature *T* ranged from 400 ${}^{\circ }$C to 1000 ${}^{\circ }$C while the melting point of a pure aluminum is known to be 660 ${}^{\circ }$C. Aluminum alloys typically demonstrate a threshold behavior [17] under laser processing. It is manifested in the reduction of the surface reflection coefficient due to intense irradiation penetration and vapor–gas channel formation. The specified threshold conditions mainly depend on the laser parameters such as power density, wavelength, and surface scanning speed, and on the material composition [18]. Two types of laser-initiated surface processes are schematically shown in Figure 1. A lightly supercritical regime is shown in Figure 1a at about equilibrium conditions, where a local inhomogeneity in target composition results in the appearance of a melted skin-layer. There is no large temperature gradient in this case. This is typical for diffusion processes of the skeleton-type cluster growth. On the other hand, to achieve the pronounced melting regime of a target, we used a 5 J pulse energy that is highly efficient for elongated dendrite-cluster growth, as one can see in Figure 1b. At the ends of the dendrite branches, one can see that B${}_{4}$C microcubes stood out (see Figure 1c).

However, the boron carbide microparticles present in AAs play a significant role in crystallization processes. The reinforcing particles act, on one hand, as thermal stoppers due to the differences in thermal properties with the AAs matrix, and, on the other hand, as barriers to the diffusion of solute at the interface between the solid and liquid phases. During the crystallization process of the composite melted pool at relatively slow cooling that is close to equilibrium, the microparticles of the reinforcing phase are pushed by growing dendrites into the inter-dendritic regions that are crystallized at last. The capture and displacement of crystalline elements by a growing dendrite cluster is a complex phenomenon, the nature of which is determined by plenty of factors. Up until now, dozens of models were developed to describe this phenomenon, as summarized in [19]. According to the analytical Kim and Rohatgi’s model [20], an outstripping of the critical crystallization velocity front would promote the capture of particles, while a velocity below the critical ones should lead to the displacement of particles by dendrites and the formation of agglomerates in the inter-dendrite regions. Thus, the value of the critical growth rate of dendrites required for a particle capture decreases with increasing size of the reinforcing particles. However, the developed models do not take to account the hydrodynamic processes that occur in the melted pool with the reinforcing particles and participate in a migration in liquid flows. Moreover, these models were originally developed for the conventional foundry and metallurgical processing. Their applicability at high local temperature gradients remains debatable. The laser action area upon melting at its energy level is mainly characterized by nonequilibrium conditions of the formation of the structure, which makes it possible to redistribute the reinforcing particles in the surface layers of the material with respect to the initial state. The velocity vector of the AA matrix melt flow near the reinforcing particle has a decisive impact [21]. It is governed by the parameters of the laser action. At this rate, during AA laser surface melting, the displacement of reinforcing elements can occur even at high speeds of crystallization front movement, speeds that significantly exceed the calculated critical values.

### 3.2. Crystal Growth Features

Crystals of convex rounded or faceted forms grow, preserving their similarity only while the crystal size does not reach a critical embryo size. This ensures the crystal shape stability. In particular, the thermo and mass-transfer deviations from a counterbalance do not exceed the equilibrium values in this case [22]. Otherwise, the crystals acquire skeletal or dendrite forms. Skeletons are the vertex and edge forms of crystals. The growth of skeletons is carried out only in the most energy-efficient directions defined by vertices and edges. Such conditions may be realized in the case of a melted media rapidly moving in the crystal growth direction. Vertices and edges parallel to the flow direction develop themselves much more efficiently than those that point in different directions. Dendrites appear as a result of the vertex and edge crystal growth that occurs during an uneven diffusion of matter to the crystal. Moreover, grain-boundary segregation of alloying and admixture elements may effectively contribute to the anisotropy effect during crystal growth [23]. A dendrite evolves starting from each trunk of the skeleton and, as a result, the appearance of branches of second, third, and higher orders occurs. Skeletons and dendrites arise due to the unstable state of the initial convex crystal form with respect to randomly occurring perturbations. The physical cause of the appearance and development of perturbations is in the spatial anisotropy of the crystal growth speed. At the first stage, the instability of growth forms and the protrusions associated with the anisotropy of the growth speed begin outpacing other areas of the interphase boundary. Further evolution of the system into a supersaturated solution leads to the start of dendrite formation. Then, each trunk of the sprawling structure grows independently from the primary protrusions and typically is thinned in its growth to the periphery upon new lateral branches forming. During the crystal growth process, crystallizing components are supplied to the phase boundary and the thermal energy is reduced during crystallization. In the course of growth from melts, mass transfer is most often carried out with the participation of forced mixing or natural convection. In an event of growth from a melted pool, the mass transfer is mostly conducted either by forced mixing or by natural convection. Nevertheless, solid surfaces, including crystalline ones, have a fixed boundary layer, in which the transfer can be considered as occurring by ordinary diffusion or thermal conductivity. However, solid surfaces and crystalline ones have a fixed boundary layer where the transfer may be caused by a diffusion or thermal conductivity. Summarizing that, dendrites are not equilibrium products of crystal growth. They are controlled by a balance of surface tension and thermodynamic driving force at the initial stage of, e.g., overcooling or oversaturation. Moreover, anisotropy has a significant impact on the resulting shape of a dendrite, which may be further transformed by diffusion processes.

### 3.3. Model of Dendrite Growth

**Figure 2:**
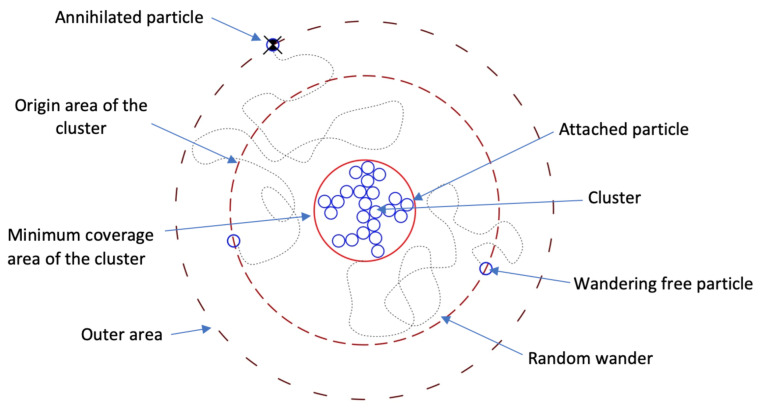
The schematic concept of the DLA growth algorithm with a minimum specified coverage area.

Based on the previous considerations, we formulated a model to simulate the initial phase of a cluster growth. We generalized the approach formulated above by introducing the anisotropy parameter and by adjusting the dimensionless latent energy, heat of transformation or anisotropy of force distributions. This generalized model allows us to predict the dendrite phenomena [24,25]. The model equations operating with dimensionless variables of the phase field p(x,y,t) and the temperature field T(x,y,t) have the following form:

(2) $$\frac{\partial T}{\partial t}=\nabla^{2}T+K\frac{\partial p}{\partial t}.$$

(3) $$\tau \frac{\partial p}{\partial t}=\frac{\partial }{\partial x}\left(\varepsilon \frac{\partial \varepsilon }{\partial \theta }\frac{\partial p}{\partial y}\right)+\frac{\partial }{\partial y}\left(\varepsilon \frac{\partial \varepsilon }{\partial \theta }\frac{\partial p}{\partial x}\right)+\nabla (\varepsilon^{2}\nabla p)+p\left(1-p\right)\left(p-1/2+m\left(T\right)\right),$$

where *K* is a dimensionless parameter that is proportional to the latent energy and inversely proportional to the cooling force; ε determines the melted layer thickness; $\varepsilon =\bar{\varepsilon }\sigma \left(\theta \right)$, where $\bar{\varepsilon }$ is the average value of ε and σ(θ) is an anisotropy; θ—an angle; τ is a positive constant of small value; and *m* sets the thermodynamic driving force as a function of temperature [26]. At this rate, the matter condition of p=0 corresponds to the liquid phase while p=1 corresponds to the solid phase. Thus, the solid–liquid phase interface is described by the last term of Equation (3). We assume the dependence m(T) in the following form: $m\left(T\right)=\frac{\alpha }{\pi }\frac{1}{\tan[\gamma \left(T-T_{e}\right)]}$, where α is a positive constant (α<1). |m(T)|<1/2 for the considered temperature range. To describe the anisotropy, the function σ(θ) is taken as $\sigma \left(\theta \right)=1+\delta \cos j(\theta -\theta_{0})$, where $\theta_{0}$ is an angle at the maximum value of ε. The parameter δ defines the strength of anisotropy, and *j* is the mode number of the anisotropy [27]. The model equations were discretized on the computational domain of a uniform grid. To solve the equations, a simple explicit scheme on a 4-point template was used [28]. The model variational parameters are dimensionless latent energy *K*, anisotropy strength δ, and anisotropy mode number *j*. Assuming these dimensionless constants given by $\bar{\varepsilon }=10^{-2};\tau =3*10^{-4};\alpha =0.9;\gamma =10;T_{e}=1$, we used a stable explicit scheme to perform the calculations. The calculation results and their discussion are given in the next section. Our approach correctly describes the growth regime, where the newly formed admixture crystallites start playing important roles in the anisotropy of the growth process. Thus, to take into account the cubic B${}_{4}$C lattices’ occurrence and its effect acting on a embryo’s shape, in the second stage, we modified the calculation algorithm with the diffusion limited agglomeration (DLA) elements. Here, the further cluster growth was modeled assuming the cluster element migration from the boundaries (schematically illustrated in Figure 2), while the calculation area was divided into subdomains: the area of cluster’s origin $R_{p}$, from where a new cluster element comes; the outer area $R_{e}$, that was located far enough from the cluster, and its role was on the element annihilation while it obtained an output; and the minimum coverage area of the cluster $R_{b}$, which covers the entire formed aggregate [29].

The adhesion probability (s) is a factor determining the dynamics of cluster growth modeling with DLA approach, where it represents the relative diffusion coefficient and takes values from S=(0;1]. Therefore, selection of the embryo shape at the initial stage of cluster growth and then the adhesive consideration also allow us to adequately describe the spatial features of the experimentally obtained clusters.

## 4. Results and Discussion

**Figure 3:**
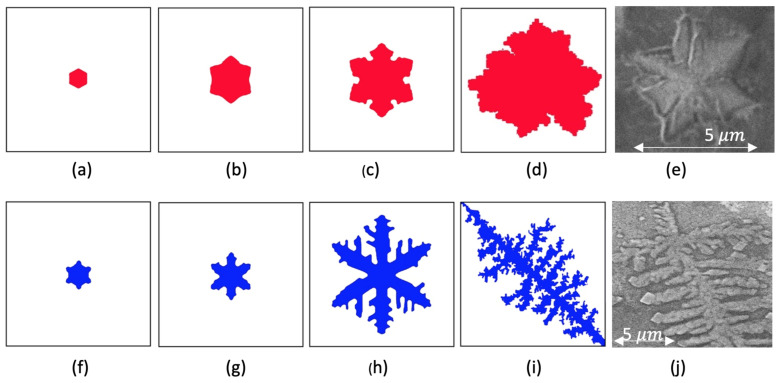
The calculation results and their comparison with real detected clusters: the top panel shows the dendrite snowflake generation at K=1.2;δ=0.050;j=6;andθ=1/2π,S=1: t = 100 (**a**), t = 300 (**b**), t = 1000 (**c**), and t = 3500 (**d**) and, corresponding to this model growth, the SEM image of a snowflake cluster formation on (**e**); the bottom panel shows dendrite star generation at K=1.6;δ=0.040;j=6;andθ=1/2π,S=1: t = 100 (**f**), t = 300 (**g**), t = 1000 (**h**), and t = 3500 (**i**) and, corresponding to this model growth, the SEM image of a star cluster on (**j**).

We localized the embryo in the center of the calculation area. Then, varying the *K*-parameter, we obtained critical embryos of different form-factors (see the embryos on Figure 3a,b,f,g). In the experiment, it depends on the temperature of the central region of the laser–target interaction and, as a consequence, depends on a thickness of the melted layer. In Figure 3, one can see the calculation results obtained for two different types of laser surface processes: local surface transformation (see the top panel of Figure 3) and cluster crystallization from a melted pool (see the bottom panel of Figure 3).

To compare the simulated clusters with the experimental ones, the fractal dimensions were estimated using the method of boxcounting [30]. The fractal dimensions *D* certify good agreement between calculation and experimental data with an accuracy of 5% for the dendrite snowflake in Figure 3d,e, with D=1.91 and for the dendrite star in Figure 3i,j, with D=1.57.

## 5. Conclusions

In conclusion, we observed two types of a clusters grow on an AA surface under different regimes of laser processing. Under the effect of melted matter of a aluminum matrix composite target, the dendrite clusters framed with B${}_{4}$C microcubes formed. The structure of the detected clusters were successfully modelled with the approach of the initial phase of a cluster growth modified with a particle’s migration effect. These results open up possibilities to fabricate a new class of alloys sculptured with refractory boron carbide microelements.

## Data Availability

The data is included in the main text.

## Abbreviations

The following abbreviations are used in this manuscript:

SEM	Scanning Electron Microscopy
NPs	Nanoparticles
HSLA	High-strength low-alloy steel
FAST	Field-assisted sintering technique
EDAX	Energy-dispersive X-ray analyzer
DLA	Diffusion-limited agglomeration
NIR	Near-infrared spectral range
